# Lateral Flow Immunoassay of SARS-CoV-2 Antigen with SERS-Based Registration: Development and Comparison with Traditional Immunoassays

**DOI:** 10.3390/bios11120510

**Published:** 2021-12-10

**Authors:** Kseniya V. Serebrennikova, Nadezhda A. Byzova, Anatoly V. Zherdev, Nikolai G. Khlebtsov, Boris N. Khlebtsov, Sergey F. Biketov, Boris B. Dzantiev

**Affiliations:** 1 A.N. Bach Institute of Biochemistry, Research Center of Biotechnology, Russian Academy of Sciences, 119071 Moscow, Russia; ksenijasereb@mail.ru (K.V.S.); nbyzova@inbi.ras.ru (N.A.B.); zherdev@inbi.ras.ru (A.V.Z.); 2 Institute of Biochemistry and Physiology of Plants and Microorganisms, Russian Academy of Sciences, 410049 Saratov, Russia; khlebtsov_n@ibppm.ru (N.G.K.); khlebtsov_b@ibppm.ru (B.N.K.); 3 Faculty of Nano- and Biomedical Technologies, Saratov State University, 410012 Saratov, Russia; 4 State Research Center for Applied Microbiology and Biotechnology, 142279 Obolensk, Moscow Region, Russia; biketov@mail.ru

**Keywords:** SARS-CoV-2, immunochromatography, test strips, surface antigen, Raman spectra

## Abstract

The current COVID-19 pandemic has increased the demand for pathogen detection methods that combine low detection limits with rapid results. Despite the significant progress in methods and devices for nucleic acid amplification, immunochemical methods are still preferred for mass testing without specialized laboratories and highly qualified personnel. The most widely used immunoassays are microplate enzyme-linked immunosorbent assay (ELISA) with photometric detection and lateral flow immunoassay (LFIA) with visual results assessment. However, the disadvantage of ELISA is its considerable duration, and that of LFIA is its low sensitivity. In this study, the modified LFIA of a specific antigen of the causative agent of COVID-19, spike receptor-binding domain, was developed and characterized. This modified LFIA includes the use of gold nanoparticles with immobilized antibodies and 4-mercaptobenzoic acid as surface-enhanced Raman scattering (SERS) nanotag and registration of the nanotag binding by SERS spectrometry. To enhance the sensitivity of LFIA-SERS analysis, we determined the optimal compositions of SERS nanotags and membranes used in LFIA. For benchmark comparison, ELISA and conventional colorimetric LFIA were used with the same immune reagents. The proposed method combines a low detection limit of 0.1 ng/mL (at 0.4 ng/mL for ELISA and 1 ng/mL for qualitative LFIA) with a short assay time equal to 20 min (at 3.5 h for ELISA and 15 min for LFIA). The results obtained demonstrate the promise of using the SERS effects in membrane immuno-analytical systems.

## 1. Introduction

The current COVID-19 pandemic has challenged the global healthcare system [1,2]. The SARS-CoV-2 virus causing this disease has four main structural proteins (spike, envelope, membrane, and nucleocapsid), which contribute to the assembly of the virus and its penetration into target cells (in the case of spike protein) [3,4]. Both spike and nucleocapsid proteins are considered antigens for the serodiagnosis of SARS-CoV-2. The incubation period for COVID-19 after the virus enters the host is estimated to be 5–6 days [5,6]. During this time, the patient is contagious, and the virus is easily transmitted from person to person by airborne droplets or direct contact.

To prevent high mortality and the risk of a severe course of the disease, timely and rapid detection of SARS-CoV-2 as well as the differential diagnoses from other coronavirus infections and influenza or SARS viruses is required [7,8,9,10]. The use of polymerase chain reaction or other amplification techniques for this purpose is associated with a long duration, specialized and expensive equipment, and skilled personnel [11,12]. Therefore, accurate point-of-care COVID-19 tests are attracting a lot of attention thanks to their speed, ease of use, and potential for diagnostic implementation. Immunoassay techniques, such as microplate enzyme-linked immunosorbent assay (ELISA) and lateral flow immunoassay (LFIA), provide more simple testing than the amplification techniques. The effectiveness of both methods for rapid mass screening of COVID-19 in routine clinical practice has been confirmed [13,14]. However, the principle of visual colorimetric detection underlining the assessment of LFIA testing results limits the sensitivity and reliability of this method. In contrast, the main disadvantage of ELISA is prolonged (several hours) testing.

To overcome the sensitivity barrier of common LFIA, the integration of surface-enhanced Raman scattering (SERS) nanotags with LFIA based on specific antigen-antibody interactions, resulting in the detection of SERS signals for quantitative interpretation of the results, was proposed [15]. The enhancing possibilities of Raman signals are attributed to the enhanced electromagnetic field near the noble metal surface under conditions of plasmon resonance [16]. According to theoretical calculations, the decrease in the detection limit provided by SERS signal enhancement can reach up to eight orders of magnitude. However, such results are rarely reached, and the typical gain in sensitivity is limited to one to two orders of magnitude [17,18]. The effectiveness of the implementation of SERS nanotags in LFIA tests for the detection of various analytes was evaluated in a review by Khlebtsov et al. [15]. The SERS-based LFIAs were successfully applied for quantitative detection of antibiotics [19], biomarkers [20,21,22,23], allergens [24], pathogens [25,26], and so on. To date, only a few researchers have proposed the integration of SERS nanotags with LFIA to improve the efficiency of COVID-19 diagnostics through serological IgM and IgG testing [18,27], whereas in the case of SARS-CoV-2 antigen detection, the SERS technique was not earlier applied.

In this study, we developed a novel SERS-based LFIA for the detection of the SARS-CoV-2 spike receptor-binding domain (RBD). A conjugate of anti-SARS-CoV-2 spike RBD antibodies with 4-mercaptobenzoic acid (MBA)-modified spherical gold nanoparticles (AuNPs) was synthesized and used as a SERS nanotag. Parameters such as the loading of the Raman reporter molecule and concentration of specific antibodies in the SERS nanotag as well as the choice of the analytical membrane for the SERS-based LFIA were optimized. After the conventional sandwich LFIA procedure, the intensity of MBA Raman scattering in the test zone was measured for quantitative assessment of the analyte. The analytical performance of SERS-based LFIA was compared with ELISA and standard AuNP-based LFIA for SARS-CoV-2 spike RBD detection using the same immunoreagents. The effectivity of SERS-based LFIA for COVID-19 diagnosis was confirmed by detecting spike RBD protein in the SARS-CoV-2 lysate. Compared to the previously mentioned LFIAs for anti-SARS-CoV-2 IgM/IgG detection, which are not recommended for use as the only diagnostic tool as well as for controlling the spread of the virus, the proposed test for determining SARS-CoV-2 Spike RBD, on the contrary, will allow for detecting the virus in the first days following infection.

## 2. Materials and Methods

### 2.1. Materials, Chemicals, and Apparatuses

Monoclonal anti-RBD antibodies (MAb), clones RBDF5 and RBDB2, and recombinant RBD were provided by HyTest (Moscow, Russia). Inactivated SARS-CoV-2 virions (2019-nCoV/Victoria/1/2020) in the form of infected Vero cells lysate were obtained from the State Research Center of Virology and Biotechnology «Vector» (Novosibirsk Region, Russia). Goat anti-mouse IgG (GAMI) antibodies were obtained from Arista Biologicals (Allentown, PA, USA). Hydrogen tetrachloroaurate(III) (HAuCl_4_), sodium citrate, sodium azide, bovine serum albumin (BSA), d-biotin-N-hydroxysuccinimide ester, dimethyl sulfoxide (DMSO), streptavidin conjugated with horseradish peroxidase (STR–HRP), 3,3′,5,5′-tetramethylbenzidine dihydrochloride (TMB), sucrose, Tris, Tween-20, Triton X-100, and 4-mercaptobenzoic acid were purchased from Sigma-Aldrich (St. Louis, MO, USA). All other chemicals were of analytical grade and used without further purification. All solutions were prepared with ultrapure water (Millipore Corporation, Burlington, MA, USA) with the resistivity of 18.2 MΩ.

The nitrocellulose membranes (CNPC-15 µm, CNPF-10 µm, and 90CNPH), conjugate fiberglass pad (PT-R7), sample pad (GFB-R4), and absorbent pad (AP045) were obtained from Mdi Easypack (Advanced Microdevices; Ambala Cantonment, Haryana, India). Absorption spectra were acquired using spectrophotometer UV-2450 (Shimadzu, Kyoto, Japan). Raman spectra were obtained using Peak seeker Pro 785 Raman spectrometer (Agiltron Inc.; Woburn, MA, USA). The 96-well transparent polystyrene microplates for ELISA were purchased from Corning Costar (Tewksbury, MA, USA). The ELISA results were measured using a Zenyth 3100 microplate spectrophotometer (Anthos Labtec Instruments; Wals, Austria). Transmission electron microscopy (TEM) images were obtained with a JEM-100C electron microscope (JEOL, Tokyo, Japan) operating at 80 kV. The nitrocellulose membranes were processed using an IsoFlow dispenser (Imagene Technology; Lebanon, NH, USA). Test strips were cut using an automatic guillotine Index Cutter-1 (A-Point Technologies; Gibbstown, NJ, USA). The intensity of the coloration was recorded using a CanoScan 9000F (Canon; Tochigi, Japan) scanner. The scanned images were digitally processed by TotalLAB software (Cleaver Scientific; Rugby, UK).

### 2.2. Biotinylation of Antibody RBDF5

Biotinylation of MAb RBDF5 was carried out as described previously [28]. The molar ratio of biotin to MAb was 10:1. A freshly prepared solution of d-biotin-N-hydroxysuccinimide ester with a concentration of 1 mM in DMSO was added to 200 μL of MAb solution with a concentration of 100 μM in 50 mM PBS containing 0.1 M NaCl (pH 7.4). The mixture was incubated for 2 h on a shaker at room temperature. Thereafter, dialysis against PBS was performed to remove the unbound low molecular weight reagents.

### 2.3. Sandwich ELISA of RBD

An amount of 100 µL of MAb RBDB2 with a concentration of 1 µg/mL in PBS was added to microplate wells. After incubation at 4 °C for 16 h and four washes by PBS containing 0.05% Triton X-100 (PBST), 100 µL of PBST solutions, containing RBD from 0.5 µg/mL to 0.5 ng/mL, were added and left to bind at 37 °C for 1 h. After the second washing step, 100 µL of biotinylated MAb RBDF5 with a concentration of 1 µg/mL was added to the wells. After incubation at 37 °C for 1 h and washing with PBST, 100 µL of STR–HRP (diluted 1:5000 with PBST) was added to the wells. Following a 1 h incubation at 37 °C, the wells were washed four times with PBST and one time with distilled water, and 100 µL of the substrate solution (0.4 mM TMB and 3 mM H_2_O_2_ in 40 mM sodium citrate buffer, pH 4.0) was added to the wells. After a further 15 min for the color development, 50 µL of 1 M H_2_SO_4_ were added to stop the reaction. The resulting optical density at 450 nm (OD450) was measured.

### 2.4. Preparation of AuNP

AuNPs were prepared using the citrate method [29]. An amount of 10 mL of a 0.01% aqueous solution of HAuCl_4_ was heated to boiling with the subsequent addition of 0.1 mL of sodium citrate under vigorous stirring. After boiling for 25 min to complete the reduction reaction, the colloidal solution was cooled to room temperature. The AuNP solution was kept in a glass bottle at 4 °C for future use.

### 2.5. Preparation of Antibody RBDF5 Conjugate with AuNP

MAb RBDF5 were conjugated with AuNPs (RBDF5–AuNP) as described by Panferov et al. [30]. Before the conjugation, the MAb were dialyzed against 10 mM Tris buffer, pH 9.0, for 1 h, at 4 °C. The pH of the AuNP solution was adjusted to 9.0 with 0.1 M K_2_CO_3_, and then the MAb were added with a concentration of 10 µg/mL. After 45 min stirring, BSA was added to the mixture at a final concentration of 0.25% in solution. The resultant mixture was centrifuged at 15,000× *g* for 15 min to remove unbound antibodies, followed by sediment resuspension in a 10 mM Tris buffer containing 1% BSA, 1% sucrose, and 0.05% NaN_3_ (pH 8.5), and storage at 4 °C.

### 2.6. Preparation of SERS Nanotag

To prepare the MBA-modified AuNPs (Au^MBA^), 1 mM MBA solution was added to 10 mL AuNPs to a final concentration in a solution of 1–10 µM. The solution was kept under constant stirring for 3 h to ensure self-assembly of the reporter molecule on the surface of the AuNP. Au^MBA^ were characterized by absorption spectra and TEM.

Anti-RBDF5 antibody-labeled Au^MBA^ (SERS nanotag) was prepared by physical adsorption of antibodies onto AuNPs. First, 10 mL of the Au^MBA^ solution was centrifuged, and the precipitate was redispersed in Milli-Q. After that, 1 mL of Au^MBA^ was adjusted to pH 8.5 with K_2_CO_3_, and 100 µL of MAb (MAb concentration was from 50 to 250 µg/mL) was added. The mixture was left under constant stirring for 3 h. Finally, 50 µL of 10% BSA was added to block nonspecific binding sites on the surface of the AuNPs. The resulting solution was left to incubate overnight. The next day, the SERS nanotag was centrifuged at 6000 rpm for 20 min at 4 °C, and the pellet was resuspended in PBS containing 5% BSA, 0.025% Tween 20, and 0.05% NaN_3_. The SERS nanotag solution was stored at 4 °C for future use.

### 2.7. Manufacturing of Tests Strips for LFIA

The nitrocellulose membranes were processed using an IsoFlow dispenser. To form the control zone (CZ), a GAMI solution with a concentration of 0.5 mg/mL in PBS was used. To form the test zone (TZ), a solution of MAb RBDB2 with a concentration of 1.0 mg/mL in PBS was used. Of each solution, 32 μL was applied per 240 mm of the width of the sheet of the nitrocellulose membranes. For standard LFIA, the conjugate RBDF5–AuNP (OD = 4.0) was sprayed onto the conjugate membrane in 400 μL per 240 mm membrane length. To form test strips for SERS-based LFIA, the SERS nanotag was applied to the conjugate membrane through the same protocol. After dispensing, all membranes were left to dry at room temperature for about 20 h. The obtained sheets with applied reactants were assembled, including the separation and absorption membranes, and were cut into strips 3.5 mm wide.

### 2.8. LFIA and Data Processing

Standard AuNP-based LFIA and SERS-based LFIA were performed at room temperature, and solutions of spike RBD protein were prepared in PBST with concentrations from 0.01 to 100 ng/mL and added to the microplate wells in a volume of 100 µL. The test strips were immersed in a vertical position with their lower end for 1 min in an aliquot of the sample, after which they were placed on a horizontal surface. The intensity of the TZ coloration was assessed after 10 min and recorded using a scanner, after which the images were digitally processed by TotalLAB software. The LFIA results were presented as the dependence of the colorimetric intensity on the log concentration to produce a sigmoidal curve.

### 2.9. SERS-Based LFIA and Data Processing

After the conventional procedure of LFIA, SERS spectra of the TZ were recorded with a Peak seeker Pro 785 Raman spectrometer (785 nm, 30 mW, the integration time was 10 s). The measurements were carried out in three independent repetitions (for three different points inside TZ and for two sets of test strips). The peak intensity of the SERS nanotag at 1076 cm^−1^ was used to quantify SESR signal. The background signal was counted as three times the standard deviation of the signal from the analytical membrane and nonspecifically adsorbed nanotags.

### 2.10. Spike RBD Protein Detection in the Lysate of SARS-CoV-2 Infected Vero Cell

The effectivity and reliability of the developed SERS-based LFIA were assessed by detecting the spike RBD protein in Vero cell lysates that contained infectious SARS-CoV-2 virions [30]. The inactivation of SARS-CoV-2 virus was performed by treatment with β-propiolactone. Since the concentration of epitopes could not be determined, solutions of the viral lysate in PBST to be analyzed were prepared by serial dilutions in the microplate wells, followed by the SERS-based LFIA procedure described in Section 2.5.

## 3. Results

### 3.1. Principle of SERS-Based LFIA for Detection of SARS-CoV-2 RBD

**Figure 1:**
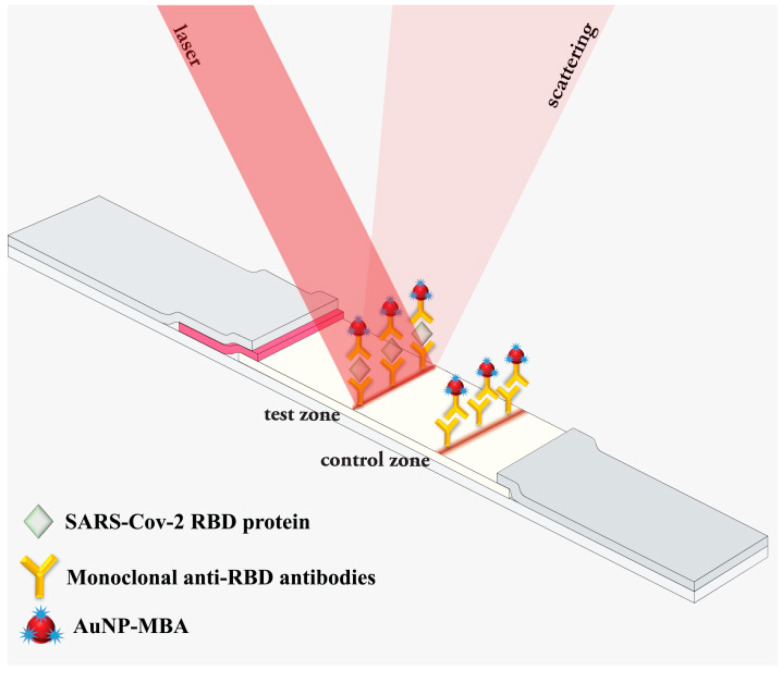
Schematic principle of SERS-based LFIA for RBD protein detection using SERS nanotag.

The principle of SERS-based LFIA depicted in Figure 1 is similar to the standard sandwich scheme of LFIA, except for the composition of the immunoconjugate (SERS nanotag in this case), which provides a detectable SERS signal for quantitative analysis of SARS-CoV-2 RBD spike protein. When the sample reached the conjugate pad, the RBD interacted with the SERS nanotag to form an immunocomplex, SERS Nanotag–RBD. The immunocomplex continued to move along the analytical membrane and was captured by immobilized anti-RBD MAb in the TZ, forming a colored band corresponding to the sandwich immunocomplex anti-RBD MAb–RBD–SERS Nanotag. The excess of SERS nanotag continued to move to the CZ, where it was captured by GAMI, forming a colored control band. Thus, the presence of the target protein in the sample results in the formation of two colored bands. Accordingly, the intensity of the characteristic peaks in the SERS spectra from the SERS nanotag is directly proportional to the protein content in the sample and can be used to graph a calibration curve for the quantitative determination of the RBD.

### 3.2. Characterization of AuNP and MBA-Modified AuNP

**Figure 2:**
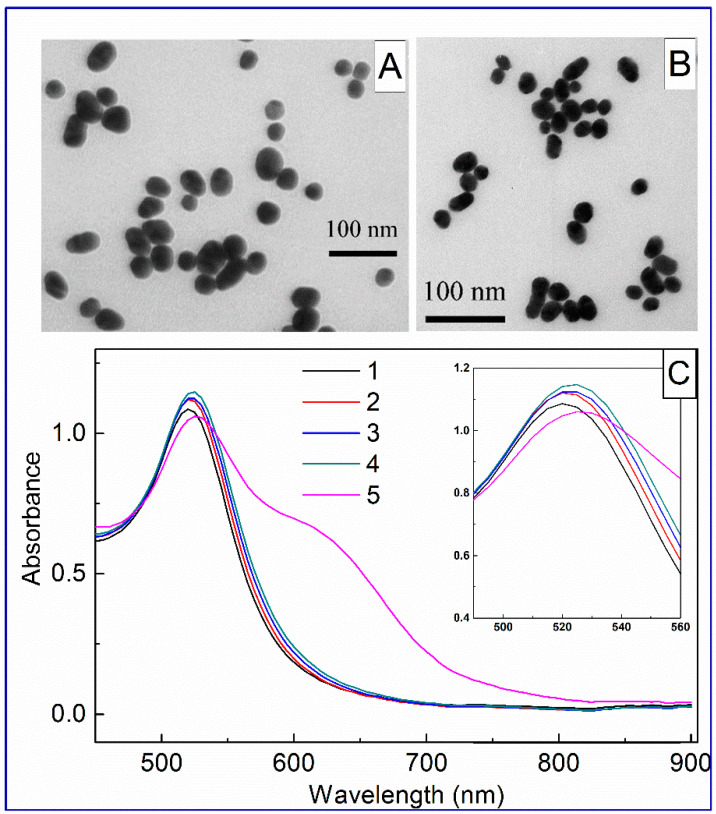
TEM image of bare AuNPs (**A**) and MBA-modified AuNP (**B**). (**C**) Absorbance spectra of bare AuNP (1) and Au^MBA^ at 1 µM (2), 3 µM (3), 5 µM (4), and 10 µM (5) concentrations. The inset shows an enlarged portion of spectra.

The structure and morphology of AuNP and Au^MBA^ were characterized by TEM and absorption spectroscopy. The TEM image revealed the monodispersed nanoparticles with a diameter distribution in the range of 24.2–40.3 nm. The average diameter of AuNPs was 31.4 ± 3.6 nm with an ellipticity of 1.1 (Figure 2A). In this study, non-covalent conjugation was preferable since this method is gentle and straightforward and allows antibodies to be immobilized on the surface of nanoparticles modified by reporter molecules with minimal conformational changes. Since 4-mercaptobenzoic acid molecules are adsorbed on the surface of AuNPs through the S-atom, and the binding of antibodies to AuNPs is dominated by electrostatic interactions between the negatively charged surface of nanoparticles promoted by carboxylic groups of MBA and antibodies containing a positive charge, it is assumed that the reporter does not significantly affect the adsorption of antibodies [31,32]. For adsorption of MBA on the surface of AuNP through thiol groups, different amounts of MBA were added to 10 mL of the colloidal solution so that its final concentration was from 1 to 10 μM. TEM measurements of AuNPs modified by 1 μM MBA demonstrated a slight increase in the average diameter up to 31.3 ± 2.9 nm with an ellipticity of 1.2 (Figure 2B). The diameter distribution was from 23.8 nm to 40.2 nm.

Figure 2C displays the spectra of nanoparticles after incubation with MBA. Bare AuNPs demonstrate surface plasmon resonance at 525 nm. AuNP solutions functionalized with 1 μM, 3 μM, and 5 μM MBA demonstrate a gradual shift in the absorption peak (insert in Figure 2C), which is associated with the formation of an MBA shell around the nanoparticles and an increase in the aggregation of nanoparticles. However, the Au^MBA^ functionalized with 10 µM MBA aggregated, as evidenced by the shift of the peak and broadening of the absorption spectrum. Therefore, further experiments continued with AuNPs containing 1 µM, 3 µM, and 5 µM MBA.

### 3.3. The Optimization of Experimental Conditions of SERS-Based LFIA

**Figure 3:**
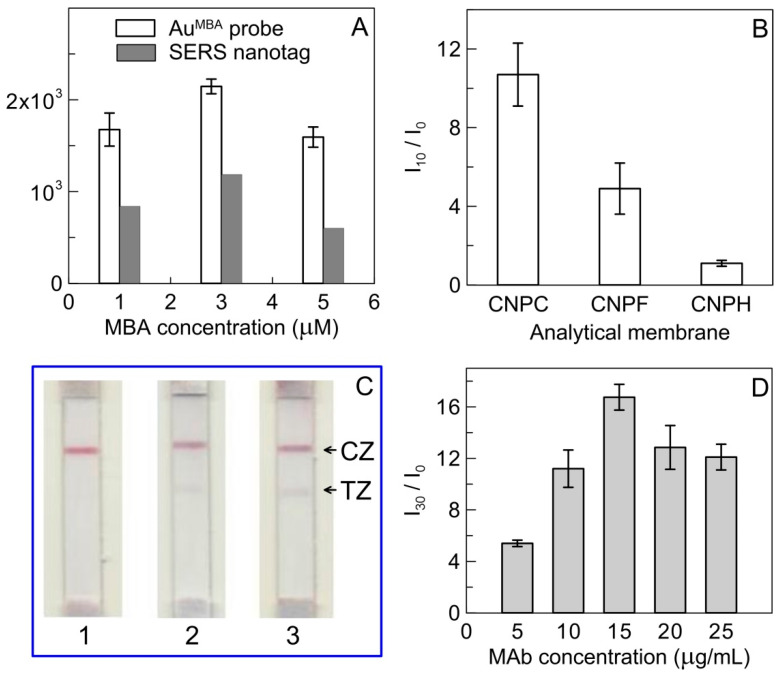
(**A**) Dependence of the Raman intensity on the concentration of MBA in the Au^MBA^ probe and SERS nanotag. (**B**) Optimization of analytical membrane. (**C**) Images of test strips after conventional procedure of LFIA using SERS nanotags with 1 µM (1), 3 µM (2), and 5 µM (3) of MBA. (**D**) Optimization of antibody concentration to prepare SERS nanotags; the positive control contains 10 or 30 ng/mL of RBD. PBS containing 1% *v/v* Tween 20 is used as a negative control.

To achieve the best performance of SERS-based LFIA, several experimental conditions were investigated. The concentrations of capture antibodies and GAMI applied to the analytical membrane to form TZ and CZ, respectively, were adjusted in a previous study [30]. In this work, the parameters that affect the performance of the SERS-based LFIA were optimized, namely, the MBA and MAb RBDF5 content in SERS nanotag and the choice of the analytical membrane. At the first stage, AuNPs modified with MBA, where the concentration of reporter molecule was 1 µM, 3 µM, and 5 µM, were obtained and 2 μL of the as-prepared Au^MBA^ probes with the AuNPs concentration of 30 nM was pipetted onto the analytical membrane in the form of a spot. Comparison of the Raman intensity of the characteristic band at 1076 cm^−1^ versus the MBA concentration, illustrated in Figure 3A, showed a higher value of Raman signal for the Au^MBA^ containing 3 μM MBA. A slight decrease in the SERS intensity when applying AuNPs modified with 5 μM MBA can be explained by the inverse effect of aggregation of nanoparticles, which leads to an unreproducible SERS signal and photodamage of analyte in the hot spot [33,34]. Further investigation of the dependences of the Raman intensity on the MBA concentration in the SERS nanotag after conjugation with MAb RBDF5, carried out as in the previous experiment, revealed similar results (Figure 3A). Therefore, for the development of the SERS-based LFIA, two SERS nanotags containing 1 μM and 3 μM of MBA were chosen.

The choice of the analytical membrane is an important step in the SERS-based LFIA development because it contributes to the flow rate of the SERS nanotag and to the binding of the SERS nanotag–RBD immunoprobe to the immobilized MAb in the TZ. The effect of three analytical membranes (namely, CNPC, CNPF, and CNPH) was investigated. The pore size and flow rate for the used analytical membranes differ at no more than one and a half times (https://mdimembrane.com/, accessed on 30 October 2021). The membranes differ also in the used additional reactants to vary protein-binding capacity, but the chemical nature of these reactants is a know-how of the manufacturer. It may be noted that the lowest colorimetric signal for membrane CNPH accords to the maximal flow rate that could be insufficient to complete the immunoreaction. The highest colorimetric signal for membrane CNPC was confirmed by the SERS measurements, according to its higher protein binding capacity than CNPF. As shown in Figure 3B, the normalized intensity (I/I_0_) of the characteristic band at 1076 cm^−1^ was significantly higher than the bands for CNPF and CNPH. Therefore, CNPC membrane was chosen for further study.

At the next stage, the selected SERS nanotags were immobilized on the conjugate pad, and the standard procedure of colorimetric LFIA was carried out. As shown in the images of the scanned test strips after analysis (Figure 3C), in the case of the SERS nanotag containing 3 μM MBA, a background signal was observed in the absence of the target RBD. Thus, the obtained results showed that the SERS nanotag containing 1 μM MBA is optimal for SERS-based LFIA.

Another important parameter affecting the sensitivity of the SERS-based LFIA is the MAb load in the SERS nanotag. In the preparation of SERS nanotag, different amounts of anti-RBDF5 MAb were added so that the final concentration of MAb in the solution was from 5 to 25 μg/mL. The effect of MAb concentration on normalized SERS intensity (I/I_0_) was investigated (Figure 3D). An increase in the normalized SERS intensity was observed with an increase in the concentration of MAb up to 15 μg/mL, whereas a further increase in a load of antibodies in the SERS nanotag was accompanied by a plateau for the dependence of I/I_0_ on MAb concentration. Thus, 15 μg/mL of MAb RBDF5 is the optimal concentration for the preparation of the SERS nanotag, which provides the highest signal-to-background ratio.

### 3.4. SERS-Based LFIA for RBD Detection

**Figure 4:**
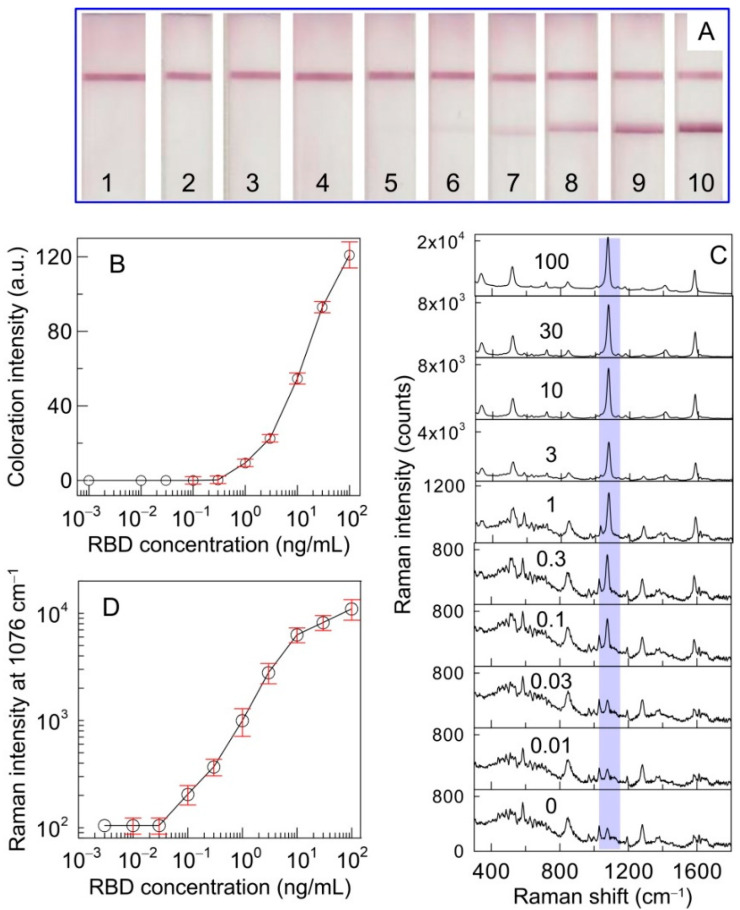
(**A**) Photographic images of LFIA strips after application of RBD at concentrations 0 (1), 0.01 (2). 0.03 (3), 0.1 (4), 0.3 (5), 1 (6), 3 (7), 10 (8), 30 (9), and 100 ng/mL (10). The top and bottom lines correspond to the control and test zones, respectively. (**B**) Calibration curve obtained after conventional LFIA procedure using SERS nanotags. The error bars indicate the STD for three measurements; (**C**) SERS spectra measured in the test line for RBD concentrations from 0.01 to 100 ng/mL. (**D**) Calibration curve of SERS-LFIA for RBD. The bars show the STD of the Raman signal at 1076 cm^−1^, measured from three independent SERS-based LFIA runs.

To assess the analytical performance of SERS-based LFIA, RBD solutions were prepared in the range from 0.01 to 100 ng/mL by diluting the analyte in PBST. After the completion of the colorimetric LFIA procedure, the color changes provided by the binding of the SERS nanotag in the TZ were observed (Figure 4A). In the colorimetric-based LFIA, a red band was visually observed where the analyte concentration was above 1 ng/mL. The quantitative assessment of colorimetric-based LFIA resulted in a 1.2 ng/mL detection limit and an operating range from 4.2 to 60.4 ng/mL (Figure 4B). The duration of the colorimetric LFIA was 15 min. Figure 4C displays the average SERS spectra acquired in the TZ for different analyte concentrations. According to the sandwich scheme of analysis, with an increase in the RBD concentration, the SERS intensity at 1076 cm^−1^ generated from the SERS nanotag gradually increases. Compared to colorimetric LFIA, characteristic MBA bands are observed in the SERS spectra without the target analyte. This phenomenon may be associated with the nonspecific binding of the SERS nanotag on the analytical membrane. Starting from the analyte concentration of 0.1 ng/mL, the characteristic peak of the SERS nanotag is reliably distinguishable from the background signal. The calibration curve represented by the dependence of the SERS signal intensity on the analyte concentration is shown in Figure 4D. The detection limit of RBD was defined as the minimum concentration at which the signal was three times the standard deviation of the background signal. The limit of RBD detection was calculated to be 0.1 ng/mL, with a working range from 0.1 to 10 ng/mL. Comparison of the results obtained by colorimetric LFIA with SERS-based LFIA for RBD detection demonstrated enhanced sensitivity by one order of magnitude. Based on the molecular weights of RBD, protein S and SARS-CoV-2 virion and S protein content in the virion [35,36,37], the given LOD value corresponds to 4 pM RBD (or protein S monomer). This concentration can be achieved in the case of complete lysis while maintaining the antigenic properties of SARS-CoV-2 virions at a concentration of 0.06 pM.

**Figure 5:**
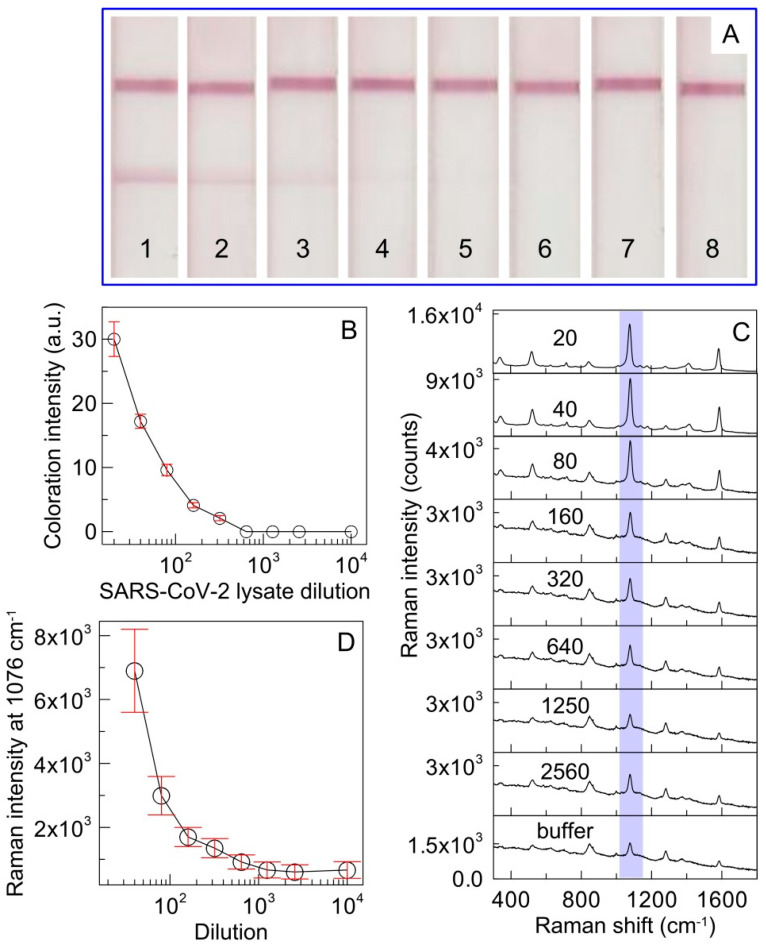
(**A**) Photographic images of LFIA strips after application of the lysate at sequential two-fold dilutions of 20 (1), 40 (2), 80 (3), 160 (4), 320 (5), 640 (6), 1280 (7), and 2560 (8); (**B**) Calibration curve obtained after conventional LFIA procedure using SERS nanotags as immunoprobe for spike RBD detection in SARS-CoV-2 viral lysate. The error bars indicate the STD for three measurements; (**C**) SERS spectra measured in the TZ for different lysate dilutions. The numbers mean the dilution. (**D**) Dependence of the SERS intensity on the lysate dilution. The bars show the STD of the Raman signal at 1076 cm^−1^, measured at five points in the middle of the test line.

The effectiveness of the developed SERS-based LFIA was evaluated in the determination of RBD protein in the SARS-CoV-2 lysate. SARS-CoV-2-inactivated virions were diluted multiple times in PBST and applied to the sample pad. Figure 5 demonstrates photographic images of test strips after completion of colorimetric LFIA procedure (Figure 5A) and compares the calibration curves obtained for colorimetric (Figure 5B) and SERS (Figure 5D) detection, respectively. The background signal consists of two sources: (1) the membrane background and (2) the signal from nonspecifically captured nanoparticles when the buffer is used instead of an analyte solution. In SERS-based LFIA for protein determination in lysate, the second contribution seems to be more intensive compared to that for RBD detection due to enhanced signal from nonspecifically captured particles. As follows from the SERS spectra acquired in the TZ after the analysis of the lysate, the SERS intensity of the band at 1076 cm^−1^ decreased with increasing sample dilution (Figure 5C). The spike protein was detected colorimetrically under conditions when the lysate was diluted 222 times, while SERS-based LFIA allowed for identifying the protein when the lysate was diluted 1250 times.

### 3.5. Comparison of SERS-Based LFIA with ELISA and Standard AuNP-Based LFIA

**Figure 6:**
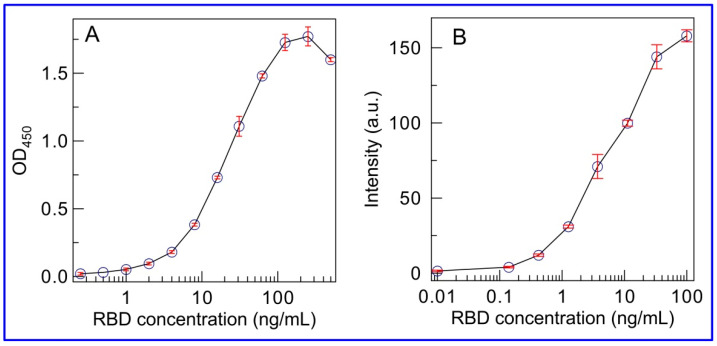
Calibration curves for spike RBD protein using ELISA (**A**) and standard AuNP-based LFIA (**B**). The error bars indicate the STD for three measurements.

Of particular interest when integrating an immunoassay with quantitative readout techniques is the comparison of the achieved analytical characteristics with those obtained by standard ELISA and AuNP-based LFIA methods. For the grounded conclusion from the experimental data, three formats of immunoassay were carried out using the same immunoreagents. Moreover, the concentration of capture MAb immobilized on the analytical membrane and detecting MAb in the labeled immunoconjugate was the same for standard LFIA and SERS-based LFIA using SERS nanotag. The calibration curve of ELISA for RBD is shown in Figure 6A. Under optimized conditions, the ELISA demonstrated a working range between 7.8 and 59.9 ng/mL with a detection limit of RBD at 0.4 ng/mL. The time of analysis was 3.5 h. After assembly of standard LFIA test strips with pre-impregnated immunocomponents, analysis of samples containing RBD in the range from 0.01 to 100 ng/mL showed a visual limit of detection at 1 ng/mL. Instrumental assessment of the LFIA results with plotting the dependence of the staining intensity of the TZ on the RBD concentration revealed a working range between 1.3 and 35.4 ng/mL with a detection limit of 0.5 ng/mL (Figure 6B). The standard qualitative AuNP-based LFIA takes 15 min. Comparing the three formats of immunoassay, it should be noted that the proposed SERS-based LFIA format combines the advantages of the considered standard ELISA and LFIA and provides an increase in the sensitivity owing to the implementation of SERS as a readout technique. Clearly, the integration of LFIA with the SERS technique involves using an additional device—a Raman spectrometer. However, today—thanks to the popularity and advantages of this method—the SERS technique is developing toward miniaturization and cost reduction. Recently, the successful development and applications of handheld Raman spectrometers were demonstrated for LFIA of various biomarkers [22,38]. The miniaturization and cost reduction of SERS-based readers makes them a promising and potential tool for developing rapid, highly sensitive, and quantitative on-site LFIA tests. In view of the aforementioned, the current study can be considered as confirmation of the prospects for further technical development of SERS-based LFIA.

## 4. Discussion

In the context of the ongoing COVID-19 pandemic, the development of new technologies for early detection of the virus and determination of the body’s response to the virus, complementing the laborious PCR testing and expanding the range of diagnostic techniques, is a priority area of research worldwide. Rapid lateral flow assay is a valuable tool for diagnosing and monitoring various diseases [39,40,41,42]. To date, rapid diagnostics of COVID-19 to determine the antigen and the presence of IgG/IgM antibodies to SARS-CoV-2 to prevent the spread of the virus is carried out using lateral flow tests [43,44]. In parallel, other methods of virus identification are being developed, including electrochemical sensors, colorimetric tests, and SERS sensors [45,46,47]. The latter has limitations associated with complex procedures for preparing the metal substrate, and in the case of direct SERS detection, with the difficulty of obtaining an intrinsic spectrum of the target analyte.

This study offers an integrated approach of lateral flow assay with a highly sensitive SERS detection, where the concentration of the analyte in one place and its highly specific determination is provided by the immunochemical principles of the analysis. The AuNP modified by MBA and conjugated with anti-RBD MAb were used as a SERS nanotag for quantitative LFIA detection. Compared to the preparation of SERS substrates, ensuring the reproducibility of SERS biosensors, the colloidal gold solution used in this work is easy to synthesize, is homogeneous, and allows for the measurements of AuNPs-based SERS nanotags on portable Raman spectrometers with high laser power and long exposure times. To achieve high analytical characteristics, the concentration of the reporter molecule and MAb in the SERS nanotag as well as the type of analytical membrane were optimized. Summarizing the results obtained, the proposed SERS-based LFIA demonstrates good performance for the detection of spike RBD protein and exhibits the effectiveness for identifying spike RBD protein in the viral lysate.

Comparison of the analytical performance of the developed SERS-based LFIA with those obtained by the membrane-based tests for detection of RBD of surface S-protein of SARS-CoV-2 is shown in Table 1. According to the overviewed membrane-based immunoassay techniques, the proposed SERS-based LFIA demonstrates comparable, and in some cases, superior performance for RBD determination.

## 5. Conclusions

In this study, the SERS-based LFIA, combining the specificity and rapidity of traditional ELISA and LFIA methods with susceptible SERS readout technique, was developed for SARS-CoV-2 spike RBD protein detection. Comparison of the three immunoassay formats revealed a decrease in the order of magnitude in the antigen detection limit after quantitative measurement of the Raman intensities of the captured SERS nanotag. The SERS-based LFIA allows for the quantitative determination of RBD in 20 min. The developed SERS-based LFIA was validated by spike RBD protein determination in inactivated SARS-CoV-2 virions. In summary, the proposed SERS-based LFIA is an alternative and complementary approach to current laboratory methods providing early, rapid, and on-site diagnosis of COVID-19.

## Figures and Tables

**Table 1:** Comparison of different SERS detection strategies for SARS-CoV-2 spike RBD protein.

Sensor	Limit of Detection	Sample	Ref.
Colloidal gold-based immunochromatographic strip	62.5 ng/mL	Standard solution	[48]
Chemiluminescence paper assay using Co–Fe@hemin-peroxidase nanozyme	0.1 ng/mL	Standard solution	[49]
LFIA using gold-enhanced AuNP	1 pg/mL	Saliva	[30]
Paper-based antigen test	0.07 nM	Standard solution	[50]
LFIA using mesoporous Si encapsulated up-conversion nanoparticles	1.6 ng/mL	Standard solution	[51]
SERS-based LFIA	0.1 ng/mL	Standard solution	This work

## Data Availability

Data are presented in the article. Initial instrumental output data are available upon request from corresponding author.

## References

[1] Gorbalenya A.E., Baker S.C., Baric R.S., de Groot R.J., Drosten C., Gulyaeva A.A., Haagmans B.L., Lauber C., Leontovich A.M., Neuman B.W. (2020). The species severe acute respiratory syndrome-related coronavirus: Classifying 2019-nCoV and naming it SARS-CoV-2. Nat. Microbiol..

[2] Synowiec A., Szczepański A., Barreto-Duran E., Lie L.K., Pyrc K. (2021). Severe acute respiratory syndrome coronavirus 2 (SARS-CoV-2): A systemic infection. Clin. Microbiol. Rev..

[3] Mariano G., Farthing R.J., Lale-Farjat S.L.M., Bergeron J.R.C. (2020). Structural characterization of SARS-CoV-2: Where we are, and where we need to be. Front. Mol. Biosci..

[4] Chen W., Feng P., Liu K., Wu M., Lin H. (2020). Computational identification of small interfering RNA targets in SARS-CoV-2. Virol. Sin..

[5] Wassie G.T., Azene A.G., Bantie G.M., Dessie G., Aragaw A.M. (2020). Incubation period of severe acute respiratory syndrome novel coronavirus 2 that causes coronavirus disease 2019: A systematic review and meta-analysis. Curr. Ther. Res. Clin. Exp..

[6] Zaki N., Mohamed E.A. (2021). The estimations of the COVID-19 incubation period: A scoping reviews of the literature. J. Infect. Public Health.

[7] Tsatsakis A., Calina D., Falzone L., Petrakis D., Mitrut R., Siokas V., Pennisi M., Lanza G., Libra M., Doukas S.G. (2020). SARS-CoV-2 pathophysiology and its clinical implications: An integrative overview of the pharmacotherapeutic management of COVID-19. Food Chem. Toxicol..

[8] Vaira L.A., Salzano G., Deiana G., De Riu G. (2020). Anosmia and ageusia: Common findings in COVID-19 patients. Laryngoscope.

[9] Bouadma L., Wiedemann A., Patrier J., Surénaud M., Wicky P.-H., Foucat E., Diehl J.-L., Hejblum B.P., Sinnah F., de Montmollin E. (2020). Immune alterations in a patient with SARS-CoV-2-related acute respiratory distress syndrome. J. Clin. Immunol..

[10] WHO (2019). Coronavirus Disease (COVID-19): Situation Report.

[11] Kanji J.N., Zelyas N., MacDonald C., Pabbaraju K., Khan M.N., Prasad A., Hu J., Diggle M., Berenger B.M., Tipples G. (2021). False negative rate of COVID-19 PCR testing: A discordant testing analysis. Virol. J..

[12] Lascarrou J.-B., Colin G., Le Thuaut A., Serck N., Ohana M., Sauneuf B., Geri G., Mesland J.-B., Ribeyre G., Hussenet C. (2021). Predictors of negative first SARS-CoV-2 RT-PCR despite final diagnosis of COVID-19 and association with outcome. Sci. Rep..

[13] Schuler C.F., Gherasim C., O’Shea K., Manthei D.M., Chen J., Giacherio D., Troost J.P., Baldwin J.L., Baker J.R. (2021). Accurate point-of-care serology tests for COVID-19. PLoS ONE.

[14] Pickering S., Batra R., Snell L.B., Merrick B., Nebbia G., Douthwaite S., Patel A., Ik M.T.K., Patel B., Charalampous T. (2021). Comparative performance of SARS-CoV-2 lateral flow antigen tests and association with detection of infectious virus in clinical specimens: A single-centre laboratory evaluation study. Lancet Microbe.

[15] Khlebtsov B., Khlebtsov N. (2020). Surface-enhanced Raman scattering-based lateral-flow immunoassay. Nanomaterials.

[16] Ding S.-Y., You E.-M., Tian Z.-Q., Moskovits M. (2017). Electromagnetic theories of surface-enhanced Raman spectroscopy. Chem. Soc. Rev..

[17] Khlebtsov B.N., Bratashov D.N., Byzova N.A., Dzantiev B.B., Khlebtsov N.G. (2019). SERS-based lateral flow immunoassay of troponin I by using gap-enhanced Raman tags. Nano Res..

[18] Chen S., Meng L., Wang L., Huang X., Ali S., Chen X., Yu M., Yi M., Li L., Chen X. (2021). SERS-based lateral flow immunoassay for sensitive and simultaneous detection of anti-SARS-CoV-2 IgM and IgG antibodies by using gap-enhanced Raman nanotags. Sens. Actuators B Chem..

[19] Fan R., Tang S., Luo S., Liu H., Zhang W., Yang C., He L., Chen Y. (2020). Duplex surface enhanced Raman scattering-based lateral flow immunosensor for the low-level detection of antibiotic residues in milk. Molecules.

[20] Hassanain W.A., Spoors J., Johnson C.L., Faulds K., Keegan N., Graham D. (2021). Rapid ultra-sensitive diagnosis of *Clostridium difficile* infection using a SERS-based lateral flow assay. Analyst.

[21] Ma Y., Liu H., Chen Y., Gu C., Wei G., Jiang T. (2020). Improved lateral flow strip based on hydrophilic−hydrophobic SERS substrate for ultra−sensitive and quantitative immunoassay. Appl. Surf. Sci..

[22] Li Y., Liu X., Guo J., Zhang Y., Guo J., Wu X., Wang B., Ma X. (2021). Simultaneous detection of inflammatory biomarkers by SERS nanotag-based lateral flow assay with portable cloud Raman spectrometer. Nanomaterials.

[23] Liu X., Yang X., Li K., Liu H., Xiao R., Wang W., Wang C., Wang S. (2020). Fe3O4@Au SERS tags-based lateral flow assay for simultaneous detection of serum amyloid A and C-reactive protein in unprocessed blood sample. Sens. Actuators B Chem..

[24] Xi J., Yu Q. (2020). The development of lateral flow immunoassay strip tests based on surface enhanced Raman spectroscopy coupled with gold nanoparticles for the rapid detection of soybean allergen β-conglycinin. Spectrochim. Acta Part A Mol. Biomol. Spectrosc..

[25] Huo C., Li D., Hu Z., Li G., Hu Y., Sun H. (2021). A novel lateral flow assay for rapid and sensitive nucleic acid detection of *Avibacterium paragallinarum*. Front. Vet. Sci..

[26] Shi L., Xu L., Xiao R., Zhou Z., Wang C., Wang S., Gu B. (2020). Rapid, quantitative, high-sensitive detection of *Escherichia coli* O157:H7 by gold-shell silica-core nanospheres-based surface-enhanced Raman scattering lateral flow immunoassay. Front. Microbiol..

[27] Liu H., Dai E., Xiao R., Zhou Z., Zhang M., Bai Z., Shao Y., Qi K., Tu J., Wang C. (2021). Development of a SERS-based lateral flow immunoassay for rapid and ultra-sensitive detection of anti-SARS-CoV-2 IgM/IgG in clinical samples. Sens. Actuators B Chem..

[28] Bayer E.A., Wilchek M. (1990). Protein biotinylation. Methods Enzymol..

[29] Wuithschick M., Birnbaum A., Witte S., Sztucki M., Vainio U., Pinna N., Rademann K., Emmerling F., Kraehnert R., Polte J.R. (2015). Turkevich in new robes: Key questions answered for the most common gold nanoparticle synthesis. ACS Nano.

[30] Panferov V.G., Byzova N.A., Biketov S.F., Zherdev A.V., Dzantiev B.B. (2021). Comparative study of in situ techniques to enlarge gold nanoparticles for highly sensitive lateral flow immunoassay of SARS-CoV-2. Biosensors.

[31] Ma W.G., Fang Y., Hao G.L., Wang W.G. (2011). Adsorption behaviors of 4-mercaptobenzoic acid on silver and gold films. Chin. J. Chem. Phys..

[32] Oliveira M.J., de Almeida P.M., Nunes D., Fortunato E., Martins R., Pereira E., Byrne H.J., Águas H., Franco R. (2019). Design and simple assembly of gold nanostar bioconjugates for surface-enhanced Raman spectroscopy immunoassays. Nanomaterials.

[33] Israelsen N.D., Wooley D., Hanson C., Vargis E. (2016). Rational design of Raman-labeled nanoparticles for a dual-modality, light scattering immunoassay on a polystyrene substrate. J. Biol. Eng..

[34] Kleinman S., Frontiera R., Henry A.-I., Dieringer J., van Duyne R. (2015). Creating, characterizing, and controlling chemistry with SERS hot spots. Phys. Chem. Chem. Phys..

[35] Ke Z., Oton J., Qu K., Cortese M., Zila V., McKeane L., Nakane T., Zivanov J., Neufeldt C.J., Cerikan B. (2020). Structures and distributions of SARS-CoV-2 spike proteins on intact virions. Nature.

[36] Huang Y., Yang C., Xu X.F., Xu W., Liu S.W. (2020). Structural and functional properties of SARS-CoV-2 spike protein: Potential antivirus drug development for COVID-19. Acta Pharmacol. Sin..

[37] Sender R., Bar-On Y.M., Gleizer S., Bernsthein B., Flamholz A., Phillips R., Milo R. (2021). The total number and mass of SARS-CoV-2 virions. Proc. Natl. Acad. Sci. USA.

[38] Xiao R., Lu L., Rong Z., Wang C., Peng Y., Wang F., Wang J., Sun M., Dong J., Wang D. (2020). Portable and multiplexed lateral flow immunoassay reader based on SERS for highly sensitive point-of-care testing. Biosens. Bioelectron..

[39] Di Nardo F., Chiarello M., Cavalera S., Baggiani C., Anfossi L. (2021). Ten years of lateral flow immunoassay technique applications: Trends, challenges and future perspectives. Sensors.

[40] Byzova N.A., Zherdev A.V., Vengerov Y.Y., Starovoitova T.A., Dzantiev B.B. (2017). A triple immunochromatographic test for simultaneous determination of cardiac troponin I, fatty acid binding protein, and C-reactive protein biomarkers. Microchim. Acta.

[41] Serebrennikova K.V., Samsonova J.V., Osipov A.P. (2019). A semi-quantitative rapid multi-range gradient lateral flow immunoassay for procalcitonin. Microchim. Acta.

[42] Wang K., Qin W., Hou Y., Xiao K., Yan W. (2016). The application of lateral flow immunoassay in point of care testing: A review. Nano Biomed. Eng..

[43] Bernasconi L., Oberle M., Gisler V., Ottiger C., Fankhauser H., Schuetz P., Fux C.A., Hammerer-Lercher A. (2020). Diagnostic performance of a SARS-CoV-2 IgG/IgM lateral flow immunochromatography assay in symptomatic patients presenting to the emergency department. Clin. Chem. Lab. Med..

[44] Basgalupp S., dos Santos G., Bessel M., Garcia L., de Moura A.C., Rocha A.C., Brito E., de Miranda G., Dornelles T., Dartora W. (2021). Diagnostic properties of three SARS-CoV-2 antibody tests. Diagnostics.

[45] Vadlamani B.S., Uppal T., Verma S.C., Misra M. (2020). Functionalized TiO2 nanotube-based electrochemical biosensor for rapid detection of SARS-CoV-2. Sensors.

[46] Büyüksünetçi Y.T., Çitil B.E., Tapan U., Anık U. (2021). Development and application of a SARS-CoV-2 colorimetric biosensor based on the peroxidase-mimic activity of γ-Fe2O3 nanoparticles. Microchim. Acta.

[47] Daoudi K., Ramachandran K., Alawadhi H., Boukherroub R., Dogheche E., Khakani M., Gaidi M. (2021). Ultra-sensitive and fast optical detection of the spike protein of the SARS-CoV-2 using AgNPs/SiNWs nanohybrid based sensors. Surf. Interfaces.

[48] Li G., Wang A., Chen Y., Sun Y., Du Y., Wang X., Ding P., Jia R., Wang Y., Zhang G. (2021). Development of a colloidal gold-based immunochromatographic strip for rapid detection of severe acute respiratory syndrome coronavirus 2 spike protein. Front. Immunol..

[49] Liu D., Ju C., Han C., Shi R., Chen X., Duan D., Yan J., Yan X. (2020). Nanozyme chemiluminescence paper test for rapid and sensitive detection of SARS-CoV-2 antigen. Biosens. Bioelectron..

[50] Hristov D., Rijal H., Gomez-Marquez J., Hamad-Schifferli K. (2021). Developing a paper-based antigen assay to differentiate between coronaviruses and SARS-CoV-2 spike variants. Anal. Chem..

[51] Guo J., Chen S., Tian S., Liu K., Ni J., Zhao M., Kang Y., Ma X., Guo J. (2021). 5G-enabled ultra-sensitive fluorescence sensor for proactive prognosis of COVID-19. Biosens. Bioelectron..

